# Does zaxinone counteract strigolactones in shaping rice architecture?

**DOI:** 10.1080/15592324.2023.2184127

**Published:** 2023-02-28

**Authors:** Jian You Wang, Justine Braguy, Salim Al-Babili

**Affiliations:** a The BioActivesLaboratory Center for Desert Agriculture, King Abdullah University of Science and Technology, Thuwal, Saudi Arabia; b Plant Science Program, Biological and Environmental Science and Engineering Division, King Abdullah University of Science and Technology (KAUST), Saudi Arabia

**Keywords:** Strigolactones, Zaxinone, Apocarotenoids, Rice (*Oryza sativa*)

## Abstract

The cleavage of plant carotenoids leads to apocarotenoids, a group of metabolites including precursors of the hormones strigolactones (SLs) and abscisic acid, regulatory and signaling molecules. Zaxinone is a recently discovered apocarotenoid growth regulator that improves growth and suppress SL biosynthesis in rice (*Oryza sativa*). To test if zaxinone also counteracts the growth regulatory effects of SLs in rice, we co-supplied zaxinone and the synthetic SL analog *rac*-GR24 to the rice SL-deficient *DWARF17* (*d17)* mutant. Results showed that co-application of GR24 and zaxinone still rescued *d17* phenotype, indicating that zaxinone and GR24 act independently in regulating root and shoot growth and development in rice.

Strigolactones (SLs) are a novel plant hormone that determines plant architecture and mediates communications in the rhizosphere.^1,2^ Structurally, SLs are carotenoid derivatives recognized by a lactone ring (D-ring, Figure1a) that is linked by an enol-ether bridge in *R*-configuration to a second moiety.^1^ While the D-ring and the enol-ether bridge are strictly conserved in all natural SLs and essential for SL activity,^3^ there is large variations in the structure of the second moiety, which is the basis for distinguishing the canonical SLs that contain a tricyclic lactone (ABC-ring) from the non-canonical ones that contain various structures as a second moiety (Figure 1a). The biosynthesis of SLs begins with the reversible isomerization of all-*trans*- into 9-*cis*-β-carotene by the isomerase DWARF27.^4,5^ Consecutive cleavage and rearrangement reactions accomplished by the Carotenoid Cleavage Dioxygenase 7 (CCD7/D17) and CCD8 (D10) lead to carlactone (CL), the central intermediate in SL biosynthesis.^2,4,6–8^ CL is the substrate of cytochrome P450 monooxygenases (CYP), including MORE AXILLARY GROWTH1 (MAX1) enzymes that belong to the CYP711A clade, which are involved in the canonical and non-canonical SL formation^9^ (Figure 1a).

**Figure 1.**
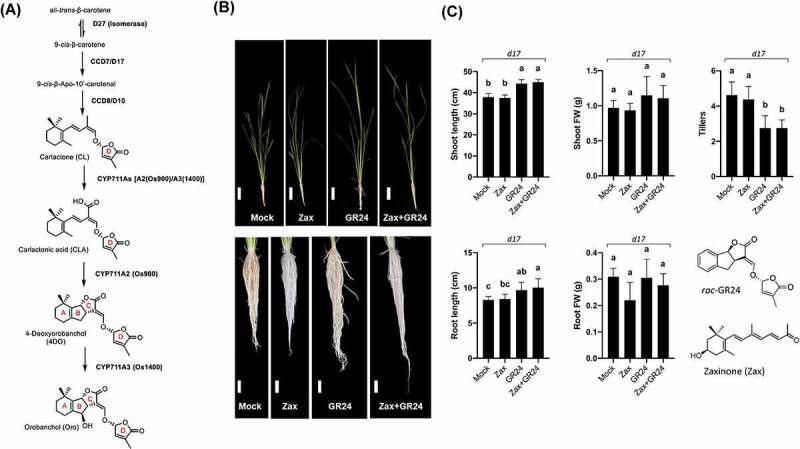
Characterization of compound effects on the rice *d17* mutant at seedling stages. (a) Proposed biosynthesis pathway of canonical SLs in rice. Abbreviations: D, *Dwarf*; CCD, *Carotenoid Cleavage Dioxygenase*; MAX, *More Axillary Growth*; Os900, OsMAX1-900; Os1400, OsMAX1-1400. (b-c) Phenotypic characterization of *d17* mutant fed with zaxinone (Zax) and *rac-*GR24. Roots of hydroponically grown *d17* mutant seedlings in the absence (Mock), presence of zaxinone (2.5 µM), *rac-*GR24 (1 µM) and of zaxinone (2.5 µM) combined with *rac*-GR24 (1 µM). Data represent the mean ± SD for 6 biological replicates. Statistical analysis was performed using one-way analysis of variance (ANOVA) and Tukey’s post hoc test. Different letters denote significant differences (*P* < .05). Scale bars at (b): up 10 cm and down 1 cm.

All types of SLs were generally considered as shoot branching inhibitors, as SL-deficient mutants are characterized by a high-branching and dwarf phenotype.^1,10,11^ However, we recently reported that canonical SLs, i.e. 4-deoxyorobanchol (4DO) and orobanchol, are important rhizospheric signals but not the predominant tillering regulators in rice (*Oryza sativa*).^12^ Consistently, the orobanchol-deficient *cyp722C* tomato (*Solanum lycopersicum*) mutant does not display high branching and dwarf phenotype.^13^ In fact, canonical SLs were originally identified as rhizospheric signals inducing seed germination of root parasitic plants and released into soil, particularly upon phosphate deficiency, to successfully recruit arbuscular mycorrhizal (AM) fungi for establishing AM symbiosis.^14–16^

The apocarotenoid growth-regulator zaxinone, produced by Zaxinone Synthase (ZAS) in rice, was identified as a negative and positive regulator of SL biosynthesis in rice and Arabidopsis, respectively.^17,18^ Interestingly, its activity in improving rice performance requires functional SL biosynthesis and signaling machinery.^17^ In addition, rice *zas* mutants showed lower tillering phenotypes accompanied by increased 4DO exudation, which could be restored by exogenous zaxinone application.^17,19^ Rice contains five MAX1 enzymes, among which Os900 mediates the formation of the rice canonical SLs, 4DO and orobanchol. So far, *max1-900* (*Os900)* mutant lines are the first rice SL-biosynthetic mutants that do not exhibit a pronounced SL-deficiency-related architectural phenotype.^12^ This observation implies that 4-DO and orobanchol are not the main regulator of rice shoot architecture and that this trait is rather determined by non-canonical SLs.^12,20^ The functionality of the SL signaling pathway in the *Os900* mutants was confirmed by zaxinone application that led to improved root and shoot growth^12^ and reduced the transcript level of SL biosynthetic genes.^17,21^ However, the question how zaxinone causes this effect on SL biosynthesis remained elusive. In this communication, we asked whether zaxinone generally acts as antagonist of SL in regulating growth and development of rice at early stages, particularly tillering.

To answer this question, we treated seedlings of the SL-deficient *d17* (*ccd7*) mutant with 2.5 μM zaxinone, 1 μM *rac*-GR24, a synthetic SL analog^1^ or with a combination of both (Figure 1b). As expected, application of GR24 alone fully rescued *d17* phenotype, increasing shoot and root length while decreasing the number of tillers. Co-application of GR24 and zaxinone led to the same effect, while application of zaxinone alone failed to rescue the *d17* phenotype (Figure 1c). The latter result confirmed that the growth-promoting activity of zaxinone depends on the presence of intact SL biosynthesis, but it also showed that zaxinone likely does not counteract the effect of SLs, as it did not affect the rescuing effect of GR24 in the *d17* mutant. This observation suggested that zaxinone might not directly interfere with SL signaling response or that the effect of SLs is much more dominant than that of zaxinone. Indeed, *rac*-GR24 also function on karrikin pathway^22^ and, possibly, the observed effects might be the results of karrikin signaling.

Notably, SL biosynthesis is barely expressed under normal growth conditions.^17^ To determine the impact of zaxinone on the expression of SL biosynthetic genes under normal conditions, we re-analyzed our related RNAseq dataset.^23^ Interestingly, none of SL biosynthesis and signaling transcripts were significantly affected by zaxinone (Figuire 2). Although we cannot rule out the possibility that zaxinone indirectly affects SL pathways *via* sugar or cytokinin metabolism under normal conditions,^23^ our data demonstrate that zaxinone does not antagonize the shoot architecture determining activity of SLs in rice, at least at the concentrations applied. Future investigations are required to shed light on the interaction between SLs and zaxinone at molecular level.

**Figure 2.**
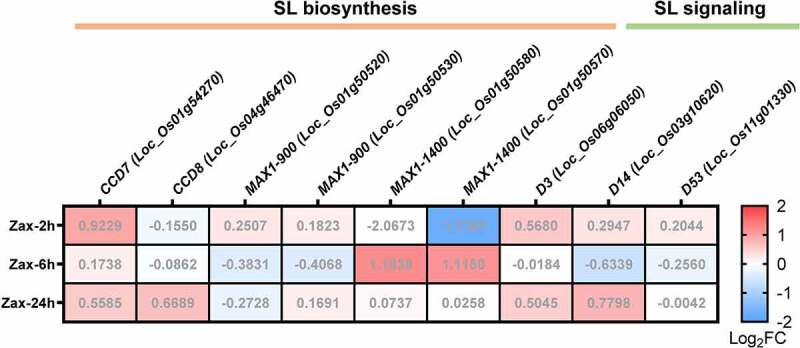
Transcriptome analysis of wild-type rice root tissues in response to zaxinone treatment at different time points. Differentially expressed genes (DEGs), following Deseq2 analysis, revealed the SL biosynthesis and signaling gene expression pattern with log_2_FoldChange (Log_2_FC). Data were extracted from Wang et al. ^23^ Numbers in the color box were Log_2_FC upon zaxinone treatment. Abbreviations: D, *Dwarf*; CCD, *Carotenoid Cleavage Dioxygenase*; MAX, *More Axillary Growth*.

## Material and methods

### Plant material

*d17*^24^ and its corresponding WT Nipponbare rice plants were surface-sterilized, cultivated, and grown under controlled conditions (a 12 h photoperiod, 200-µmol photons m^−2^ s^−1^ and day/night temperature of 27/25°C) according to the published protocol.^25^ 7-day-old seedlings were transferred into black falcon tubes filled half-strength modified Hoagland nutrient solution (nutrient compositions listed in^25^) with adjusted pH to 5.8 with KOH.

### Exogenous applications of zaxinone and GR24

For investigating the effect of zaxinone and GR24 on *d17* seedlings, 7-day-old rice seedlings were grown hydroponically in 1/2 Hoagland nutrient solution containing 2.5 µM zaxinone (obtained from Buchem B.V.; Apeldoorn, The Netherlands), 1 µM *rac*-GR24 (purchased from StrigoLab; Turin, Italy), or the corresponding volume of the solvent (mock; acetone) for 2 weeks. The solution was changed twice per week, adding the chemical at each renewal.

## RNA-seq data analysis

RNAseq dataset was extracted from Wang et al. ^23^ and the reads were aligned to the *O. sativa* genome v7.0 (http://phytozome.jgi.doe.gov/; Phytozome v12.1). Data processing and analysis were performed using the LSTrAP workflow,^26^ and differential gene expression (DGE), read counts from HTSeq, were analyzed by DESeq2.^27^

## Data Availability

All data generated or analyzed during this study are included in this published article.

## References

[1] Al-Babili S, Bouwmeester HJ. Strigolactones, a novel carotenoid-derived plant hormone. Annu Rev Plant Biol. 2015;66:161–4. doi:10.1146/annurev-arplant-043014-114759. 25621512

[2] Wang JY, Lin P-Y, Al-Babili S, et al. (2021a). On the biosynthesis and evolution of apocarotenoid plant growth regulators. Seminars in Cell and Developmental Biolology 109, 3–11. doi: 10.1016/j.semcdb.2020.07.007 32732130

[3] Yoneyama K, Xie X, Yoneyama K, Kisugi T, Nomura T, Nakatani Y, Akiyama K, McErlean CSP. Which Are the Major Players, Canonical or Non-Canonical Strigolactones? J Exp Bot. 2018;69(9):2231–2239. 10.1093/jxb/ery090. 29522151

[4] Alder A, Jamil M, Marzorati M, Bruno M, Vermathen M, Bigler P, Ghisla S, Bouwmeester H, Beyer P, Al-Babili S, et al. The path from β-carotene to carlactone, a strigolactone-like plant hormone. Science. 2012;335(6074):1348–1351. doi:10.1126/science.1218094. 22422982

[5] Abuauf H, Haider I, Jia K-P, Ablazov A, Mi J, Blilou I, Al-Babili S. The arabidopsis DWARF27 gene encodes an all-trans-/9-cis-β-carotene isomerase and is induced by auxin, abscisic acid and phosphate deficiency. Plant Science. 2018;277:33–42. doi:10.1016/j.plantsci.2018.06.024. 30466598

[6] Bruno M, et al. On the substrate-and stereospecificity of the plant carotenoid cleavage dioxygenase 7. FEBS Lett. 2014;588:1802–1807. doi:10.1016/j.febslet.2014.03.041. 24685691

[7] Bruno M, et al. Insights into the formation of carlactone from in-depth analysis of the CCD 8-catalyzed reactions. FEBS Lett. 2017;591:792–800. doi:10.1002/1873-3468.12593. 28186640

[8] Chen G-TE, et al. 9‑cis‑β‑Apo‑10ʹ‑carotenal is the precursor of strigolactones in planta. Planta. 2022;256:88. doi:10.1007/s00425-022-03999-9. 36152118

[9] Zhang Y, van Dijk ADJ, Scaffidi A, Flematti GR, Hofmann M, Charnikhova T, Verstappen F, Hepworth J, van der Krol S, Leyser O, et al. Rice cytochrome P450 MAX1 homologs catalyze distinct steps in strigolactone biosynthesis. Nat Chem Biol. 2014;10(12):1028–1033. doi:10.1038/nchembio.1660. 25344813

[10] Umehara M, et al. Inhibition of shoot branching by new terpenoid plant hormones. Nature 455. 2008;195-200.e 10:1186. 10.1038/nature07272 18690207

[11] Gomez-Roldan V, Fermas S, Brewer PB, Puech-Pagès V, Dun EA, Pillot J-P, Letisse F, Matusova R, Danoun S, Portais J-C, et al. Strigolactone inhibition of shoot branching. Nature. 2008;455(7210):189–194. doi:10.1038/nature07271. 18690209

[12] Ito S, Braguy J, Wang JY, Yoda A, Fiorilli V, Takahashi I, Jamil M, Felemban A, Miyazaki S, Mazzarella T, et al. Canonical strigolactones are not the major determinant of tillering but important rhizospheric signals in rice. Science Advances. 2022;8(44):eadd1278. doi:10.1126/sciadv.add1278. 36322663 PMC9629705

[13] Wakabayashi T, et al. Direct Conversion of Carlactonoic Acid to Orobanchol by Cytochrome P450 CYP722C in Strigolactone Biosynthesis. Science Advances 5 (12): eaax9067. 2019. doi:10.1126/sciadv.aax9067. PMC6989309 32064317

[14] Akiyama K, Matsuzaki K-I, Hayashi H. Plant sesquiterpenes induce hyphal branching in arbuscular mycorrhizal fungi. Nature. 2005;435(7043):824–827. doi:10.1038/nature03608. 15944706

[15] Lanfranco L, Fiorilli V, Venice F, Bonfante P. Strigolactones cross the kingdoms: plants, fungi, and bacteria in the arbuscular mycorrhizal symbiosis. J Exp Bot. 2018;69(9):2175–2188. doi:10.1093/jxb/erx432. 29309622

[16] Fiorilli V, Wang JY, Bonfante P, Lanfranco L, Al-Babili S. Apocarotenoids: old and new mediators of the arbuscular mycorrhizal symbiosis. Front Plant Sci. 2019;10:1186. doi:10.3389/fpls.2019.01186. 31611899 PMC6776609

[17] Wang JY, Haider I, Jamil M, Fiorilli V, Saito Y, et al. The apocarotenoid metabolite zaxinone regulates growth and strigolactone biosynthesis in rice. Nat Commun. 2019;10:810. doi:10.1038/s41467-019-08461-1. 30778050 PMC6379432

[18] Ablazov A, Mi J, Jamil M, Jia K-P, Wang JY, Feng Q, Al-Babili S. The Apocarotenoid Zaxinone Is a Positive Regulator of Strigolactone and Abscisic Acid Biosynthesis in Arabidopsis Roots. Front Plant Sci. 2020;11:578. doi:10.3389/fpls.2020.00578. 32477389 PMC7240130

[19] Ablazov A, Votta C, Fiorilli V, Wang JY, Aljedaani F, Jamil M, Balakrishna A, Balestrini R, Liew KX, Rajan C, et al. ZAXINONE SYNTHASE 2 regulates growth and arbuscular mycorrhizal symbiosis in rice. Plant Physiol. 2023;191(1):382–399. doi:10.1093/plphys/kiac472. 36222582 PMC9806602

[20] Wang JY, Braguy J, Chen GTE, Jamil M, Balakrishna A, Berqdar L, Al-Babili S. Perspectives on the metabolism of strigolactone rhizospheric signals. Front Plant Sci. 2022a;13:1062107. doi:10.3389/fpls.2022.1062107. 36507392 PMC9729874

[21] Wang JY, Jamil M, Lin P-Y, Ota T, Fiorilli V, Novero M, Zarban RA, Kountche BA, Takahashi I, Martínez C, et al. Efficient mimics for elucidating zaxinone biology and promoting agricultural applications. Mol Plant. 2020;13(11):1654–1661. doi:10.1016/j.molp.2020.08.009. 32835886 PMC7656291

[22] Carbonnel S, Torabi S, Gutjahr C. MAX2 -independent transcriptional responses to rac- GR24 in Lotus japonicus roots. Plant Signal Behav. 2021;16:1. doi:10.1080/15592324.2020.1840852. PMC7781757 33126824

[23] Wang JY, Alseekh S, Xiao T, Ablazov A, Perez de Souza L, Fiorilli V, Anggarani M, Lin P-Y, Votta C, Novero M, et al. Multi-omics approaches explain the growth-promoting effect of the apocarotenoid growth regulator zaxinone in rice. Communications Biology. 2021b;4(1):1222. doi:10.1038/s42003-021-02740-8. 34697384 PMC8545949

[24] Butt H, Jamil M, Wang JY, Al-Babili S, Mahfouz M. Engineering plant architecture via CRISPR/Cas9-mediated alteration of strigolactone biosynthesis. BMC Plant Biol. 2018;18(1):1–9. doi:10.1186/s12870-018-1387-1. 30157762 PMC6116466

[25] Wang JY, Chen GTE, Jamil M, Braguy J, Sioud S, Liew KX, Balakrishna A, Al-Babili S. Protocol for characterizing strigolactones released by plant roots. STAR Protocols. 2022b;3(2):101352. doi:10.1016/j.xpro.2022.101352. 35620066 PMC9127222

[26] Proost S, Krawczyk A, Mutwil M. LSTrAP: efficiently combining RNA sequencing data into co-expression networks. BMC Bioinform. 2017;18(1):444. doi:10.1186/s12859-017-1861-z. PMC5634843 29017446

[27] Love MI, Huber W, Anders S. Moderated estimation of fold change and dispersion for RNA-seq data with DESeq2. Genome Biol. 2014;15:550. doi:10.1186/s13059-014-0550-8. 25516281 PMC4302049

